# Supplementary material for: A synaptic mechanism for encoding the learned value of action-derived safety
Source: bioRxiv. 2025 Aug 4:2025.08.04.668532. Preprint. [Version 1] doi: 10.1101/2025.08.04.668532 (PMC12340825; doi:10.1101/2025.08.04.668532)
Supplement: Supplement 1 [file media-1.pdf]

Supplementary Figures and Table

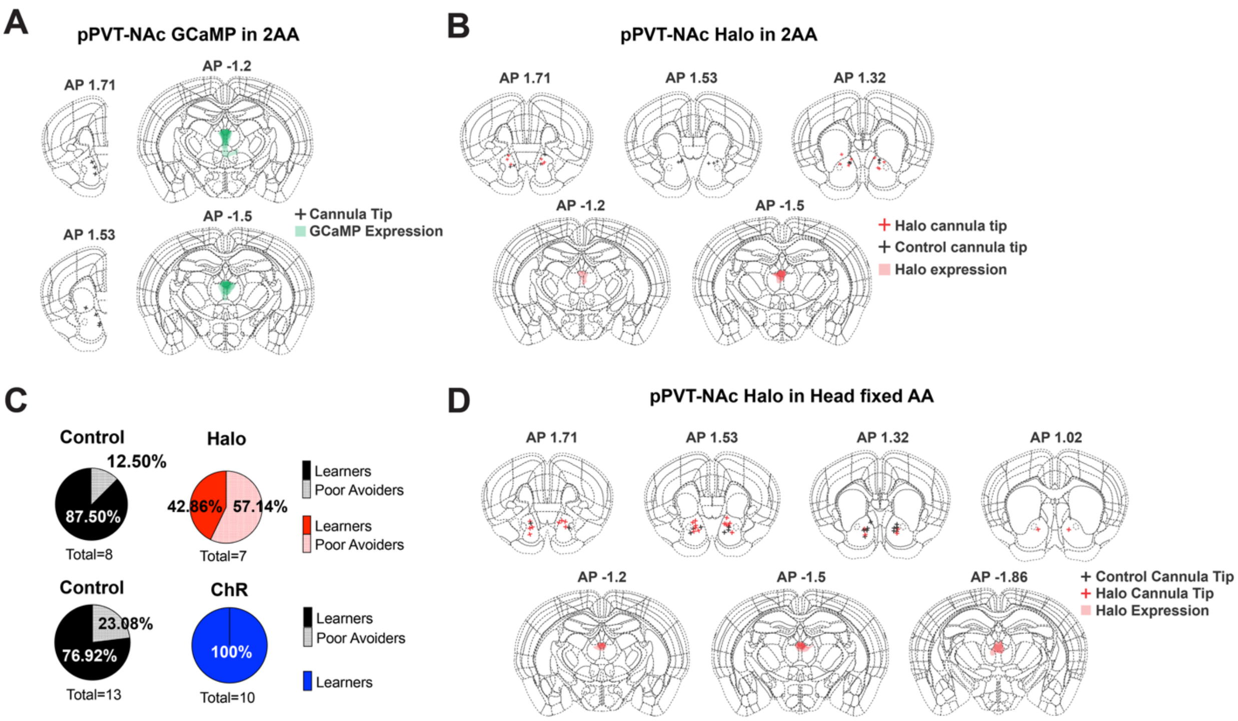

Supplementary Figure 1: Related to Figure 1. (A) Map of optic fiber cannula tip locations for photometry experiment in figures 1A-G (left). Map of viral spread of GCaMP at bregma -1.2 and -1.5 (right). **(B)** Map of optic fiber cannula tip locations for optogenetics experiment in figures 1H-L (top). Map of viral spread of halorhodopsin (Halo) at bregma -1.2 and -1.5 (bottom). **(C)** Proportion of poor avoiders (<30% avoidance day 3 of training) per optogenetic experimental group in figures 1H-L and S3. **(D)** Map of optic fiber cannula tip locations for optogenetics experiment in figures 1M-O (top). Map of viral spread of halorhodopsin at bregma -1.2 and -1.5 (bottom).

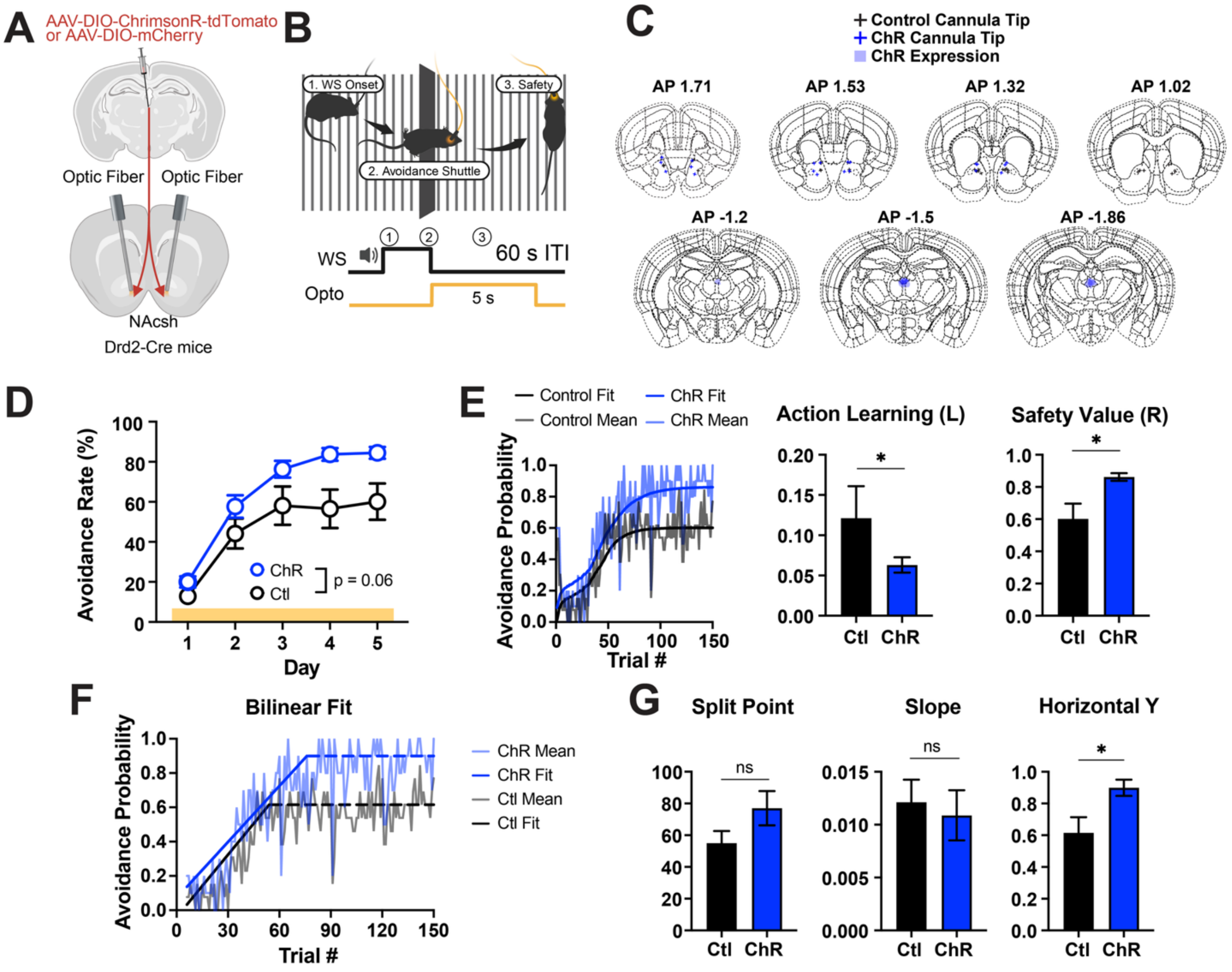

Supplementary Figure 2: pPVT-NAc activation boosts safety value. (A) Drd2-Cre mice were injected with ChrimsonR (ChR) or fluorescent control in pPVT to bilateral stimulate terminals in NAc. **(B)** Schematic of closed-loop optogenetic manipulation in the 2AA task (see Methods). **(C)** Map of optic fiber cannula tip locations (top). Map of viral spread of ChrimsonR at bregma -1.2, -1.5, and -1.86 (bottom). **(D)** Performance in the 2AA task with excitation of pPVT-NAc at safety (Control n=13, ChR n=10 mice). 2-way Anova group effect: $p=0.0554$. **(E)** Avoidance probability across trial with best fit Rescorla-Wagner model probability by group (left). Best fit learning and value parameters by group with bootstrap estimated error (middle, right). Bootstrap permutation test for group difference: Learning $p=0.0412$; Value $p=0.0208$. **(F)** Avoidance probability across trial with best fit bilinear model by group. **(G)** Bilinear model best fit parameters by group with bootstrap estimated error. Bootstrap permutation test for group difference: Split point $p=0.084$; Slope $p=0.7284$; Horizontal $p=0.0188$. Data are shown as mean ± s.e.m. *$p < 0.05$, ns: $p > 0.05$.

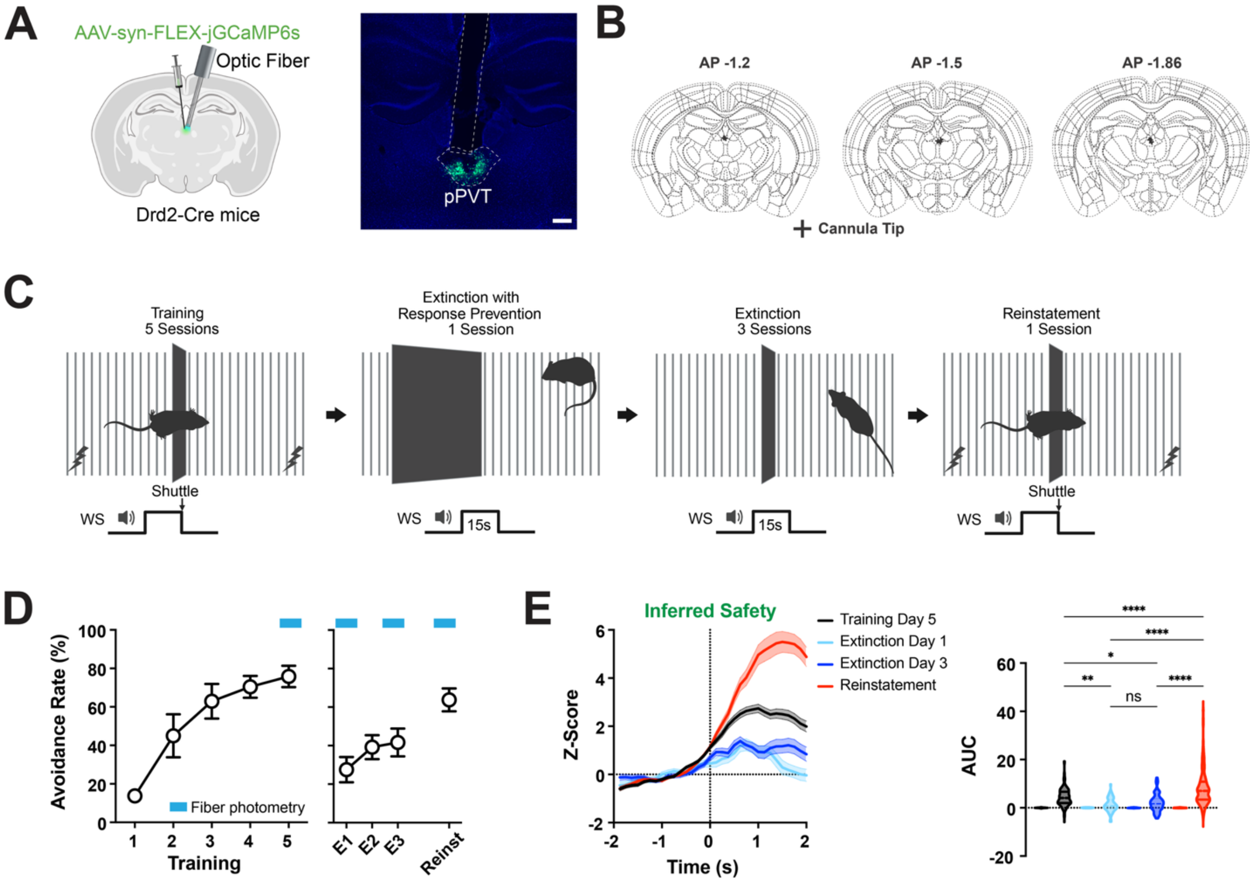

Supplementary Figure 3: pPVT tracks safety devaluation. (A) Drd2-Cre mice were injected with GCaMP to record during 2AA and extinction (left). Representative image of injection and recording site (right). Scale bar, 200um. **(B)** Map of optic fiber cannula tip locations. **(C)** Schematic of training protocol (see Methods). **(D)** Performance in the 2AA task (n=8 mice). **(E)** GCaMP dF/F z-score normalized at shuttle (safety) with optogenetic inhibition by day of training (left). Quantification of post event activity (right). AUC, pairwise comparisons between groups, linear mixed-effects model for repeated measures: see statistics table. Data are shown as mean ± s.e.m. *$p < 0.05$, **$p < 0.01$, ****$p < 0.0001$, ns: $p > 0.05$.

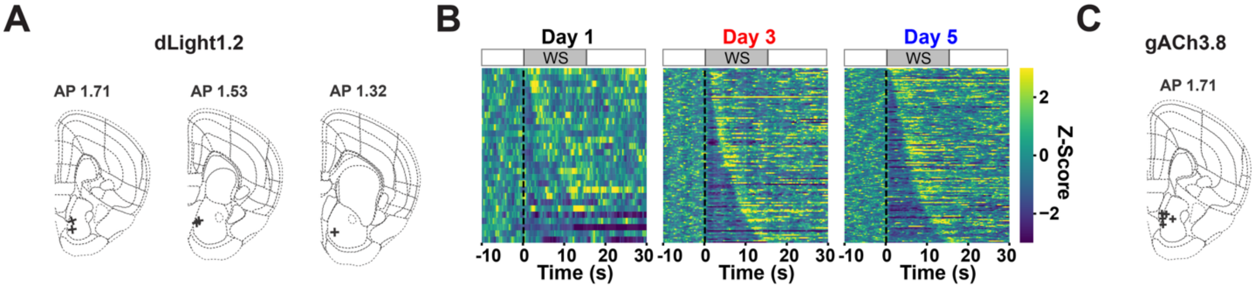

Supplementary Figure 4: Related to figures 2 and 3. (A) Map of fiber optic cannula tip location for dLight photometry experiment in figures 2A-F. **(B)** Heatmaps of dLight signals during avoidance trials, ordered by avoidance latency (low to high). Related to figures 2A-F. **(C)** Map of fiber optic cannula tip location for gACh photometry experiment in figures 3D-J.

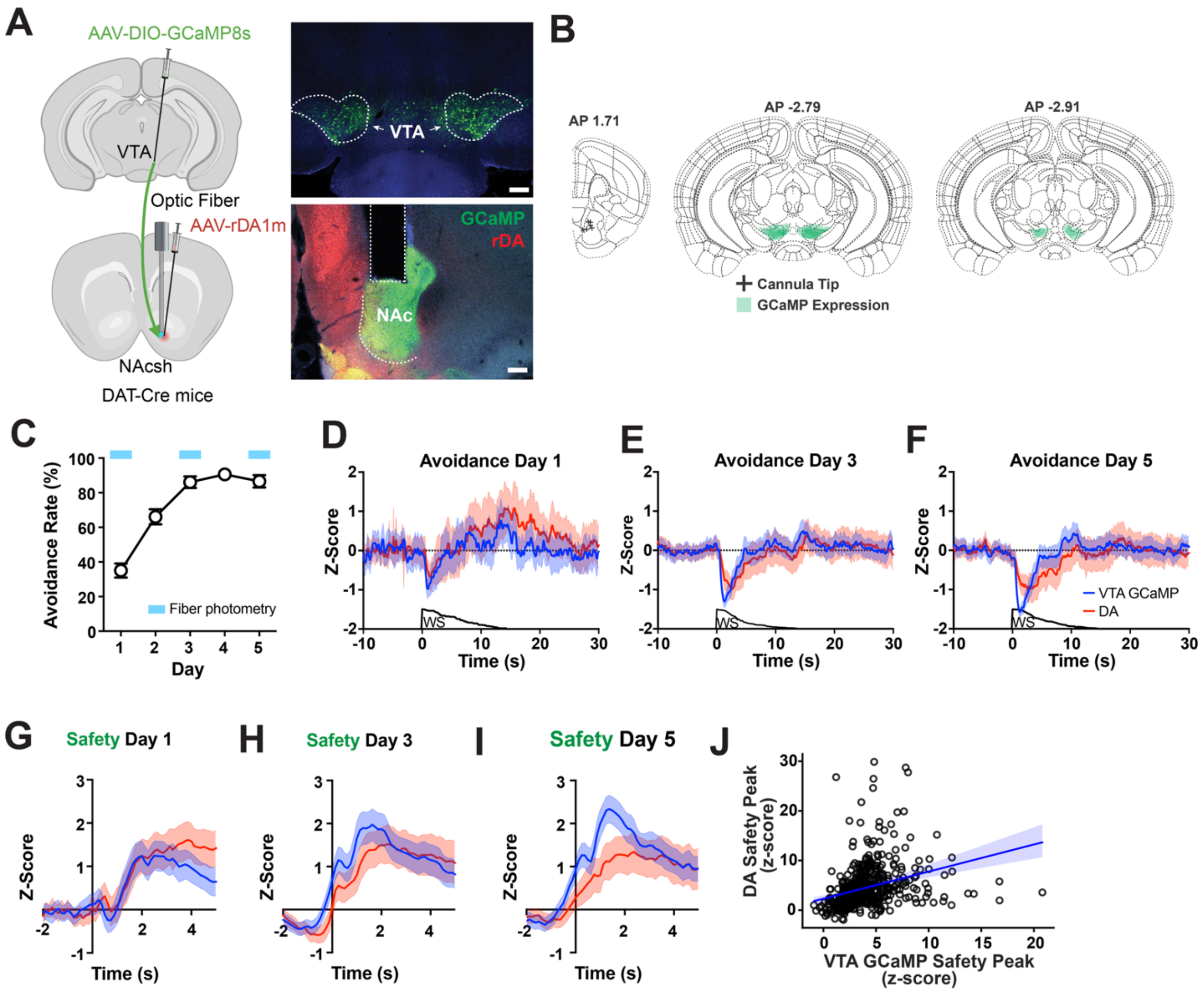

Supplementary Figure 5: rDA1m sensor activity matches dopaminergic terminals. (A) DAT-Cre mice were injected with GCaMP8s in the VTA and rDA1m in the NAc to validate the GRAB DA sensor (left). Representative images of injection and recording sites (right). Scale bars, 200um. **(B)** Map of optic fiber cannula locations (left) and GCaMP viral expression in VTA at bregma -2.79 and -2.91 (right). **(C)** Performance in the 2AA task (n=6 mice). **(D-F)** GCaMP (blue) and rDA (red) dF/F z-score normalized in all avoidance trials on training days 1 (**D**), 3 (**E**) and 5 (**F**), with average WS duration below. **(G-I)** dF/F z-score normalized at safety on training days 1 (**G**), 3 (**H**) and 5 (**I**). **(J)** Correlation between peak z-score value at safety by trial of GCaMP and rDA. Pearson correlation: $p<0.0001$. Data are shown as mean ± s.e.m.

A

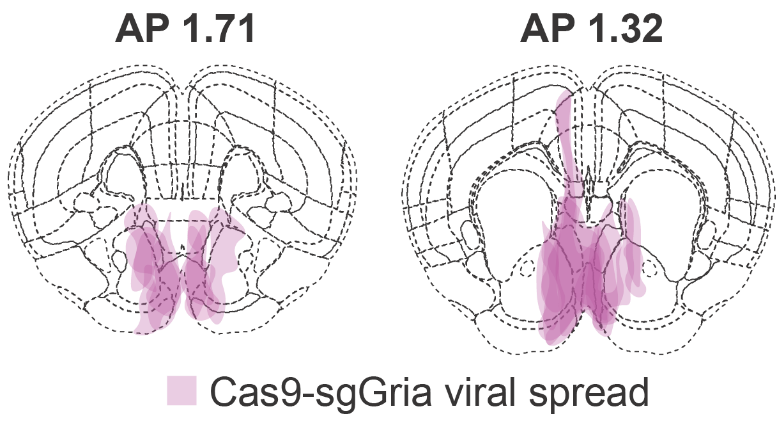

Supplementary Figure 6: Related to figure 5. (A) Map of viral spread for sgGria KO in figures 5N-Q at bregma 1.71 and 1.32.

Supplementary Table 1: Statistics Table

Figure	Comparison	Statistical Test	P Value
**Figure 1F**	Shuttle Initiation AUC Day Effect	AUC~C(Day)+(1|Subject) F(2, 285) = 3.512	* p=0.0311
	Day 1 vs Day 3	Pairwise comparison Sidak corrected	p=0.9937
	Day 1 vs Day 5	Pairwise comparison Sidak corrected	p=0.3913
	Day 3 vs Day 5	Pairwise comparison Sidak corrected	* p=0.0324
**Figure 1G**	Safety AUC Day Effect	AUC~C(Day)+(1|Subject) F(2, 398) = 5.700	* p=0.0036
	Day 1 vs Day 3	Pairwise comparison Sidak corrected	p=0.7459
	Day 1 vs Day 5	Pairwise comparison Sidak corrected	* p=0.0209
	Day 3 vs Day 5	Pairwise comparison Sidak corrected	* p=0.0161
**Figure 1J**	Day x Group	2 Way ANOVA F (5, 65) = 1.780	p=0.1294
	Day	F (2.758, 35.86) = 13.16	**** p<0.0001
	Group	F (1, 13) = 5.573	* p=0.0345
**Figure 1L**	Learning Ctl vs Halo	Bootstrap permutation test LCtl-LHalo=-0.096341, 95% CI5000=[-0.12019125,0.1031405]	p=0.1032
	Value Ctl vs Halo	Bootstrap permutation test RCtl-RHalo=0.34851, 95% CI5000=[-0.33509075,0.3382775]	* p=0.0448
**Figure 1N**	First day of training avoidance	Unpaired t test t=1.387, df=26	p=0.1771
	Last day of training avoidance	Unpaired t test t=1.178, df=26	p=0.2493
**Figure 1O**	Ctl Laser Off vs On	Paired t test t=0.7042, df=12	p=0.4947
	Halo Laser Off vs On	Paired t test t=3.458, df=15	** p=0.0035
**Figure 2E**	Shuttle Initiation AUC Day Effect	AUC~C(Day)+(1|Subject) F(2, 302) = 2.001	p=0.1370
**Figure 2F**	Safety AUC Day Effect	AUC~C(Day)+(1|Subject) F(2, 201) = 1.929	p=0.1479
**Figure 2I**	Day x Group	2 Way ANOVA F (8, 52) = 0.3850	p=0.9238
	Day	F (2.094, 27.22) = 57.84	**** p<0.0001
	Group	F (2, 13) = 0.6944	p=0.517
**Figure 2K**	Day	AUC~Day*Group+(1|Subj) F(2, 574) = 10.488	**** p<0.0001
	Group	F(1, 574) = 4.742	* p=0.0298
	Day x Group	F(2, 574) = 0.457	p=0.6336

	Ctl vs Halo Day 1	Pairwise comparison Sidak corrected	p=0.0857
	Ctl vs Halo Day 3	Pairwise comparison Sidak corrected	** p=0.0075
	Ctl vs Halo Day 5	Pairwise comparison Sidak corrected	* p=0.0259
**Figure 3C**	DA Amplitude Baseline vs DHBE	Paired t test t=24.68, df=4	**** p<0.0001
**FIgure3I**	Shuttle Initiation AUC Day Effect	AUC~C(Day)+(1|Subject) F(2, 217) = 4.073	* p=0.0183
	Day 1 vs Day 3	Pairwise comparison Sidak corrected	p=0.4551
	Day 1 vs Day 5	Pairwise comparison Sidak corrected	* p=0.0182
	Day 3 vs Day 5	Pairwise comparison Sidak corrected	p=0.2072
**Figure 3J**	Safety AUC Day Effect	AUC~C(Day)+(1|Subject) F(2, 345) = 5.337	** p=0.0052
	Day 1 vs Day 3	Pairwise comparison Sidak corrected	p=0.1370
	Day 1 vs Day 5	Pairwise comparison Sidak corrected	** p=0.0036
	Day 3 vs Day 5	Pairwise comparison Sidak corrected	p=0.2902
**Figure 3O**	Training	AUC~Day*Treatment*Time + (1|Subject) F(1, 312) = 5.129	* p=0.0242
	Treatment	F(1, 312) = 14.011	*** p=0.0002
	Time	F(1, 312) = 1.126	p=0.2894
	Treatment x Time	F(1, 312) = 7.007	** p=0.0085
	Training x Time	F(1, 312) = 2.557	p=0.1108
	Treatment x Training	F(1, 312) = 3.084	p=0.0800
	Treatment x Training x Time	F(1, 312) = 1.545	p=0.2149
	Pre vs On Pre-Training Saline	Pairwise comparison Sidak corrected	p=0.3277
	Pre vs On Pre-Training CNO	Pairwise comparison Sidak corrected	** p=0.0089
	Pre vs On Post-Training Saline	Pairwise comparison Sidak corrected	p=0.0708
	Pre vs On Post-training CNO	Pairwise comparison Sidak corrected	p=0.9668
	On Pre-Training Saline vs Pre-Training CNO	Pairwise comparison Sidak corrected	p=0.9027
	On Pre-Training Saline vs Post-Training Saline	Pairwise comparison Sidak corrected	**** p<0.0001
	On Pre-Training Saline vs Post-Training CNO	Pairwise comparison Sidak corrected	p=0.9772
	On Pre-Training CNO vs Post-Training Saline	Pairwise comparison Sidak corrected	**** p<0.0001

	On Pre-Training CNO vs Post-Training CNO	Pairwise comparison Sidak corrected	p=0.2119
	On Post-Training Saline vs Post-Training CNO	Pairwise comparison Sidak corrected	** p=0.0018
**Figure 4E**	AMPA Currents	Unpaired t test t=3.241, df=39	** p=0.0024
**Figure 4F**	AMPA Rise Time	Unpaired t test t=2.683, df=39	* p=0.0107
**Figure 4G**	AMPA Decay Time	Unpaired t test t=3.193, df=39	** p=0.0028
**Figure 4H**	NMDA Currents	Unpaired t test t=1.446, df=39	p=0.1562
**Figure 4I**	AMPA/NMDA Ratio	KS normality test: yoked, trained	** p=0.002, *** p=0.0006
	AMPA/NMDA Ratio	Mann Whitney test U=118	* p=0.0168
**Figure 4J**	Paired-pulse Ratio	Unpaired t test t=3.775, df=39	*** p=0.0005
**Figure 4K**	Input x Treatment	2way ANOVA F (4, 76) = 7.266	*** p<0.0001
	Input	F (4, 76) = 701.3	*** p<0.0001
	Treatment	F (1, 19) = 8.625	** p=0.0085
	70mV	Bonferroni's multiple comparisons test	**** p<0.0001
	Rectification Index	Unpaired t test t=2.257, df=19	* p=0.0360
**Figure 4L**	Inhibition by NASPM	Unpaired t test t=5.545, df=10	*** p=0.0002
**Figure 5E**	AMPA Currents	Unpaired t test t=4.257, df=22	*** p=0.0003
**Figure 5F**	AMPA Rise Time	Unpaired t test t=2.494, df=22	* p=0.0206
**Figure 5G**	AMPA Decay Time	Unpaired t test t=2.288, df=22	* p=0.0321
**Figure 5H**	Paired-pulse Ratio	Unpaired t test t=3.825, df=22	*** p=0.0009
**Figure 5J**	NMDA Currents	Unpaired t test t=0.7933, df=19	p=0.4374
**Figure 5K**	AMPA/NMDA Ratio	Unpaired t test t=2.421, df=19	* p=0.0256
**Figure 5M**	Inhibition by NASPM	Unpaired t test t=3.173, df=9	* p=0.0113
**Figure 5O**	Day x Group	Mixed effects F (4, 40) = 2.140	p=0.0936
	Day	F (1.645, 16.45) = 52.67	**** p<0.0001
	Group	F (1, 10) = 5.082	* p=0.0478
**Figure 5Q**	Learning Empty vs sgGria1	Bootstrap permutation test LCtl-LHalo=-0.0137, 95% CI5000=[-0.01905325,0.01904415]	p=0.2136
	Value Empty vs sgGria1	Bootstrap permutation test RCtl-RHalo=0.1512, 95% CI5000=[-0.1499,0.1458]	* p=0.0372
**Figure S2D**	Day x Group	2way ANOVA F (5, 105) = 1.987	p=0.0865
	Day	F (2.631, 55.24) = 77.02	**** p<0.0001
	Group	F (1, 21) = 4.116	p=0.0554
**Figure S2E**	Learning Ctl vs ChR	Bootstrap permutation test LCtl-LChR=0.05827, 95% CI5000=[-0.0645,0.0565]	* p=0.0412

	Value Ctl vs ChR	Bootstrap permutation test RCtl-RChR=-0.26011, 95% CI5000=[-0.2347,0.2493]	* p=0.0208
**Figure S2G**	Split point	Bootstrap permutation test sCtl-sChR=-22.0, 95% CI5000=[-25.0,21.0]	p=0.084
	Slope	Bootstrap permutation test aCtl-aChR=0.001246, 95% CI5000=[-0.0060,0.0064]	p=0.7284
	Horizontal Y	Bootstrap permutation test f(ts)Ctl-f(ts)ChR=-0.283716, 95% CI5000=[-0.1943,0.2385]	* p=0.0188
**Figure S3E**	Shuttle AUC Day Effect	AUC~C(Day)+(1|Subject) F(3.0, 490) = 29.413	**** p<0.0001
	TD5 vs. ExD1	Pairwise comparison Sidak corrected	** p=0.0031
	TD5 vs. ExD3	Pairwise comparison Sidak corrected	* p=0.0237
	TD5 vs. Reinst	Pairwise comparison Sidak corrected	**** p<0.0001
	ExD1 vs. ExD3	Pairwise comparison Sidak corrected	p=0.9421
	ExD1 vs. Reinst	Pairwise comparison Sidak corrected	**** p<0.0001
	ExD3 vs. Reinst	Pairwise comparison Sidak corrected	**** p<0.0001
**Figure S5I**	Safety Peak GCaMP vs DA	Pearson r=0.3160	**** p<0.0001
